# Effective Management of Leptospirosis: A Case Report of Combined Doxycycline and Ceftriaxone Therapy

**DOI:** 10.7759/cureus.67909

**Published:** 2024-08-27

**Authors:** Chetan A Timande, Deepika R Kanyal, Aditya K Bhargava, Shantanu R Sabale, Sanket Mahajan, Sudhanshu M Dakre

**Affiliations:** 1 Hospital Administration, Datta Meghe Institute of Higher Education and Research, Wardha, IND; 2 Clinical Embryology, Datta Meghe Institute of Higher Education and Research, Wardha, IND

**Keywords:** ceftriaxone, doxycycline, antibiotic therapy, bacterial infection, leptospirosis

## Abstract

Leptospirosis is a bacterial infection caused by the pathogen Leptospira. The disease is primarily transmitted through contact with animals (mainly rats) or through exposure to contaminated water or soil. Underdeveloped countries and places with poor housing and sanitation are at higher risk. Leptospirosis often presents with nonspecific symptoms, making it difficult to diagnose. This can delay the initiation of appropriate treatment. In the case presented, the patient had a history of cough, high fever, and a rash over various parts of the body. This combination of respiratory symptoms, systemic fever, and dermatological manifestations led to the suspicion of an infection.

Initial blood examinations revealed a significantly increased white blood cell (WBC) count, indicating an infection. Further enzyme-linked immunosorbent assay (ELISA) testing was confirmed by active immunoglobulin M (IgM) antibodies specific to leptospira species, followed by a chest X-ray scan. The antibiotics doxycycline (for seven days) and ceftriaxone (two weeks) were used to treat the leptospirosis. The patient was also given antipyretics to bring down fever and antitussive agents to suppress the cough. Hydration and breathing exercises were also given high priority in healing from this illness. After treatment, the patient did very well, he sweated less before dawn, the rash started to go away, and finally, even coughing was controlled.

Further blood tests have shown that now the WBC is in the normal range and the IgM antibody level has dropped. In other words, the infection has been eradicated. For detailed information, refer to the case study "A Moral Call," which argues that early treatment and intervention are critical in managing leptospirosis. Getting this severe infection with early antibiotic therapy and nursing care gives people a chance for complete recovery from their illness. There must be more studies into this disease's long-term effects and how to prevent it when the risk group is more significant.

## Introduction

Leptospirosis, caused by the bacteriumLeptospira, is a widespread zoonotic disease that can adversely affect both domestic animals and humans. The infection can occur through exposure of the skin surfaces, particularly of the nose, eyes, and mouth, to contaminated water or soil. Additionally, human leptospirosis may spread indirectly or through direct contact with infected animal urine and tissues [1]. This disease is a significant global health concern, often presenting with nonspecific symptoms such as high fever, headache, myalgia, and rash, which can progress to more severe conditions like kidney damage, liver failure, and respiratory impairment [2]. Early diagnosis is crucial to prevent severe outcomes and to enable effective treatment for populations at risk of exposure to contaminated environments [3].

In this report, we discuss a noteworthy case of leptospirosis involving a 24-year-old patient who presented with a week-long history of persistent cough, high fever, and a rash spread throughout the body. Differential diagnosis included ruling out other causes of fever and rash such as dengue, malaria, typhoid fever, and viral exanthems through appropriate laboratory tests. The patient also experienced chills and weight loss during the onset of illness. Laboratory examinations, including a complete blood count (CBC), revealed an elevated white blood cell (WBC) count, and enzyme-linked immunosorbent assay (ELISA) confirmed the presence of Immunoglobulin M (IgM) antibodies specific toLeptospiraspecies. The patient was treated with a combination of doxycycline and ceftriaxone recommended by a medical practitioner. Doxycycline is effective against a wide array of pathogens, including atypical bacteria, its broad-spectrum activity is beneficial, and the primary relevance in this case is its proven efficacy specifically against Leptospira species, while ceftriaxone is effective against *Streptococcus pneumoniae*, *methicillin-susceptible staphylococci*,* Haemophilus influenzae*, *Moraxella catarrhalis*, and *Neisseria species*. Ceftriaxone plus doxycycline is typically suggested as initial empiric antibiotic therapy for hospitalized patients, though significant diversity in prescribing practices exists. This combination ensures comprehensive coverage during the initial treatment phase, which is crucial for managing severe leptospirosis and its potential complications [4-8].

After a few days of treatment, the patient showed significant improvement, with subsided fever and diminished cough. Follow-up CBC and ELISA indicated normalization of the WBC along with that IgM antibody for leptospira was absent, suggesting successful treatment of the infection. This case highlights the importance of timely diagnosis and intervention in managing leptospirosis. Early antibiotic therapy and preventive care are essential to ensure successful patient recovery. Further exploration into long-term outcomes and treatment strategies for leptospirosis is warranted.

## Case presentation

Patientinformation

A 24-year-old male was referred to emergency in our hospital in Wardha, Maharashtra. The patient came with no significant medical history, but in the past seven days, he had complained of insidious throat pain, intermittent fever, and cough. Informed consent was obtained from the patient prior to collecting and using his medical information for this study, ensuring that he understood the nature and purpose of the data collection, as well as his right to confidentiality and privacy.

Physicalexamination

The patient presented with a high fever along with progressive body chills. He also had a productive cough and cold alongside chest pain. The chest pain was due to a prolonged cough with sputum. The patient’s temperature was 39.44°C and upon auscultation, it was found that the patient had decreased air entry on the right side of the lung. On further physical examination, the patient was found to have a rash all over the abdominal area, the patient also had weight loss.

Diagnosis

Differential diagnosis included ruling out other causes of fever and rash such as dengue, malaria, typhoid fever, and viral exanthems through appropriate laboratory tests. His diagnostic examination shows an elevated WBC count of 18500 per cu.mm as shown in Table 1 and ELISA Leptospira IgM reactive.

**Table 1:** Investigation from admission and days later WBC: white blood cell

Values				Reference value
	Day 1	Day 2	Day 3	Normal range
WBC	18500/cu.mm	110000/cu.mm	9000/cu.mm	4500 to 11000/cu.mm
Platelet	2.57 Lacs/cu.mm	2.44 Lacs/cu.mm	4.22 Lacs/cu.mm	1.5 to 4.5 Lacs/cu.mm
Lymphocytes	20% of total WBC	25% of total WBC	35% of total WBC	Adults: 20% to 40% of total WBC, pediatrics: 60% to 90% of total WBC
Hemoglobin	15 mg	12.9 mg	15.6 mg	Male: 13.8 to 17.2 g/dL, female: 12.1 to 15.1 g/dL
Red cell distribution width	14.4 fL	14.6 fL	14.1 fL	11.8 to 14.5 fL

**Figure 1:**
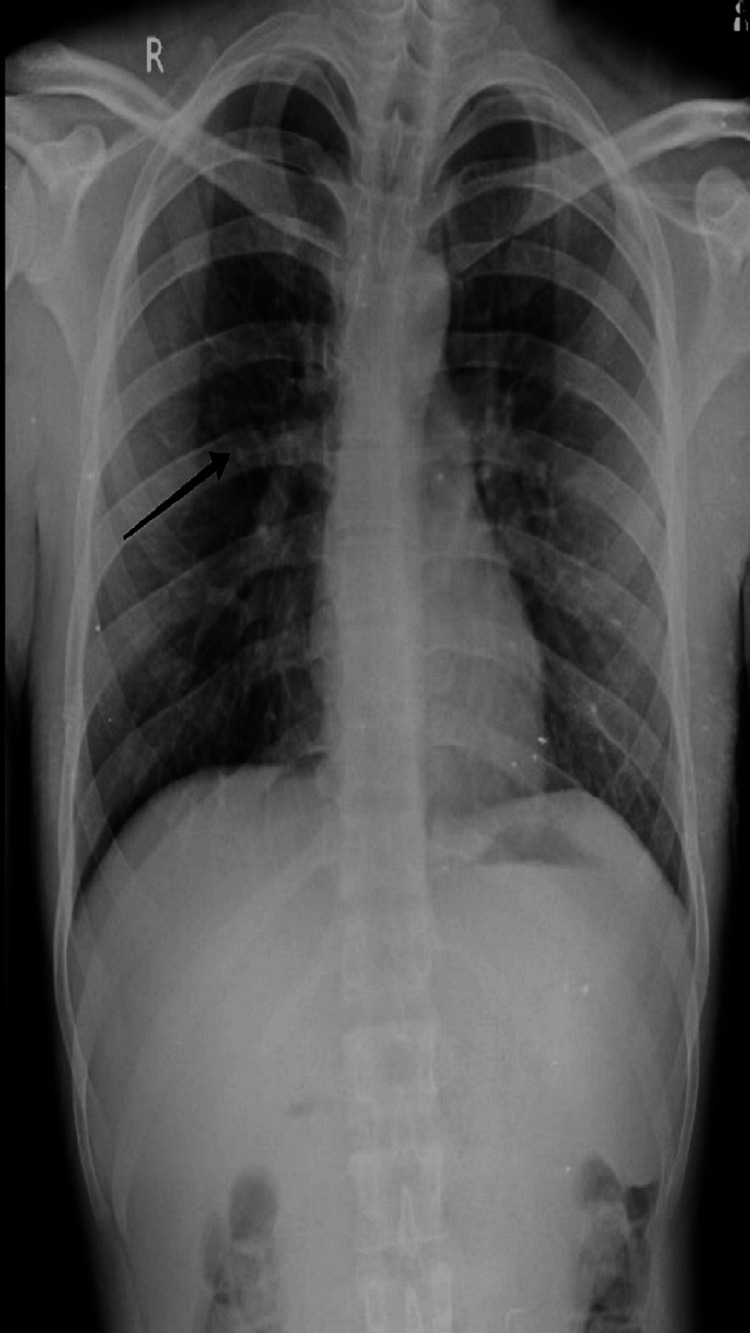
Chest X-ray showing pneumonia infection

The patient was vitamin-B12 deficient at 159 pg/mL. A chest X-ray was also done and hazy opacities within the bilateral lower lung field were seen. The diagnostic results from ELISA and radiological intervention clearly suggest leptospira infection and lower lobe pneumonia. Considering that the infection had spread, antibiotic therapy was ordered for a week to cure the disease as shown in Figure 1.

**Figure 2:**
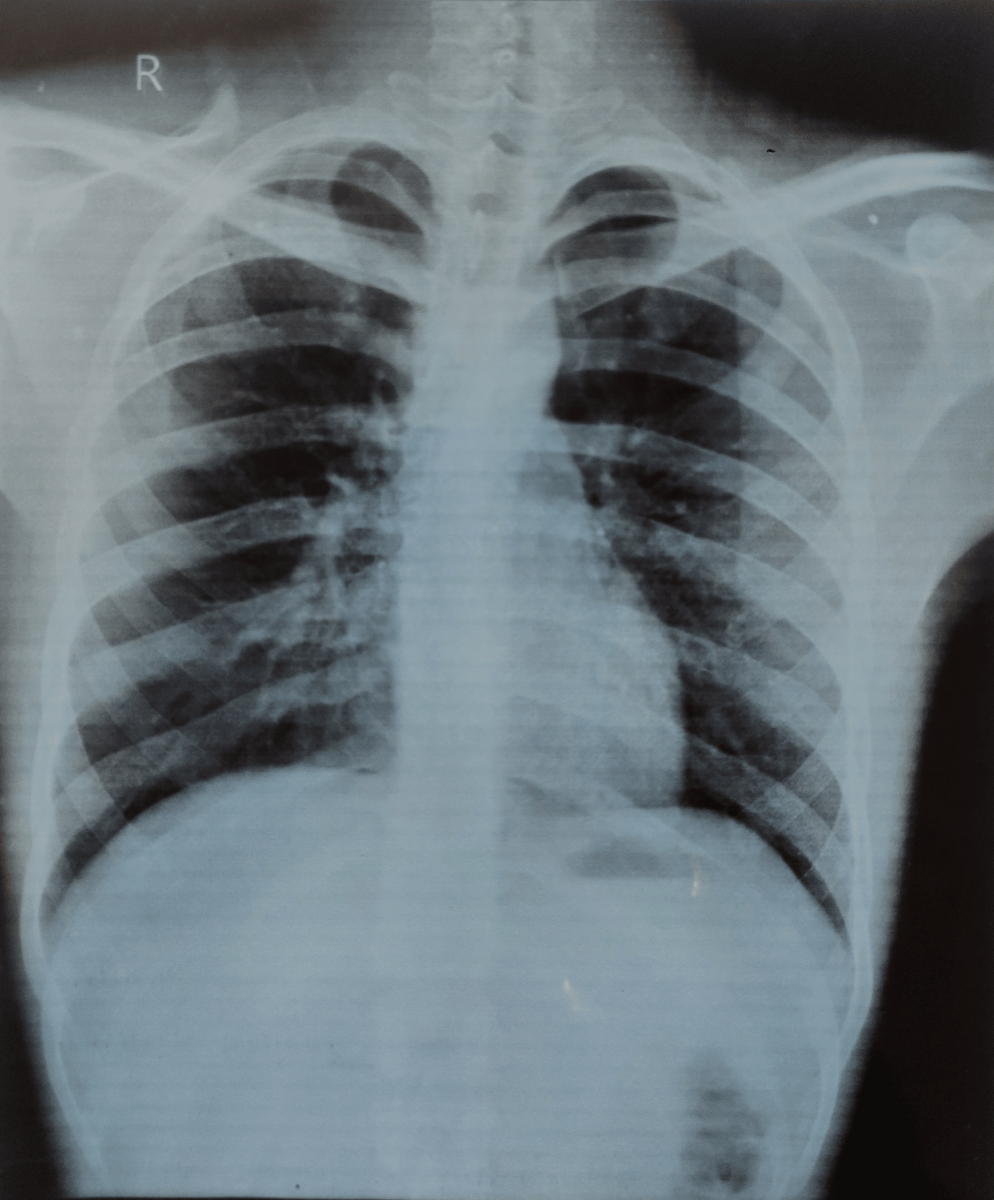
Post-treatment X-ray scan

The patient was treated with a seven-day therapeutic regimen with ceftriaxone 1 g intravenously, twice daily, and doxycycline 100 mg intravenously, twice daily. Ceftriaxone and doxycycline combination targets the bacterial agent effectively, aiming to eradicate the *leptospira* pathogen and stop disease progression. Supportive medication includes pantoprazole 40 mg intravenously, once daily to prevent gastrointestinal problems commonly associated with antibiotic use, and ondansetron 4 mg intravenously, as needed as antiemetic relief against nausea and vomiting. Paracetamol 650 mg orally, three times daily helps with fever and pain, along with ambroxol thrice a day to suppress cough symptoms. Montelukast and levocetirizine were also prescribed to alleviate respiratory symptoms and provide relief from allergic reactions optineurin one ampule diluted in 100 mL of normal saline once daily was also given intravenously to support neurological function and maintain electrolyte balance. A series of CBCs had shown continuous normalization of WBC refer to Table 1. A repeated ELISA *leptospira* antibody test had shown complete eradication of the infection and a repeat radiographic chest X-ray also suggested clear lungs as shown in Figure 2. After successfully completing the seven-day antibiotic treatment and management some symptoms may take longer to fully resolve, the patient was discharged. The patient continued to be monitored and received supportive care post-discharge to ensure complete recovery.

Follow-up

After discharge, the patient was prescribed paracetamol twice daily along with montelukast for five days. Continuation of steam inhalation with betadine gargles three times a day was also recommended. The patient has been set for a follow-up seven days post-discharge in the outpatient department. After receiving a diagnosis, the patient felt anxious about the severity of symptoms. Still, there was some relief in knowing the specific cause of the health issue. It was also a significant advantage for the patient to have as much information discussed by the team as possible. Following the treatment, one could identify the enhancement in the form of less severe fever, rash, and controlled cough. The patient has appreciated the medical care provided to him

## Discussion

In this study, a male with leptospirosis was treated with antibiotic therapy of doxycycline and ceftriaxone to see the result. Our study found that doxycycline and ceftriaxone regimen successfully cured the leptospirosis infection.

Phimda K et al., have described the effectiveness of doxycycline against leptospirosis compared to azithromycin, which is more expensive. This makes doxycycline not only a therapeutically effective but also a financially sustainable option, especially in resource-constrained conditions where the burden of leptospirosis is often highest, whereas for the treatment of severe leptospirosis, ceftriaxone, and sodium penicillin g were equally efficient [6]. Once-daily treatment and the increased spectrum of ceftriaxone against microorganisms provide additional benefits over intravenous penicillin [7].

Naing et al., have done a meta-analysis on the efficacy of antibiotics on leptospirosis. Seven randomized controlled studies were identified for this study. These random controlled trials examined the efficacy of antibiotics such as doxycycline for the treatment of human Leptospirosis. These studies made comparisons between antibiotics (i.e., an antibiotic versus an alternative antibiotic) in the primary trial and a placebo, except for cephalosporin. The article emphasizes the efficacy of doxycycline. In 92 out of 976 leptospirosis patients, 27 investigations on Jarisch-Herxheimer reaction (JHR) reported the onset of reaction within one to 48 hours of the first antibiotic dose being administered [8].

There is a growing understanding of the necessity for standardized treatment regimens, as evidenced by the numerous biological researches examining the effects of different parameters on doxycycline's efficacy in treating leptospirosis. Among these parameters, the time and dosage of doxycycline administration continue to be discussed. To optimize the therapeutic advantages of doxycycline, some researchers advise administering it as soon as possible. However, other researchers stress the significance of customizing the dosage according to the unique characteristics of each patient and the severity of their illness. In research by Phimda K et al., significant reductions in symptom severity and duration were reported following a regimen of doxycycline administration in leptospirosis patients, further supporting its efficacy [9,10]. 

## Conclusions

The case presented has helped the patient in the treatment of the leptospirosis. The antibiotic therapy of doxycycline and ceftriaxone cured the patient successfully, as confirmed by the CBC reports and ELISA tests conducted after administering the medication. To verify the findings of the study, we encouraged conducting additional studies with a bigger sample size, as this article was based on only one patient.
